# The effects of lithium on cognition in humans: A systematic review

**DOI:** 10.1177/02698811251371139

**Published:** 2025-10-17

**Authors:** Talitha Najmillah Sabtiari, Samuel Myrtle, Stelios Orfanos, Allan H. Young, Rebecca Strawbridge

**Affiliations:** 1 Department of Psychological Medicine, Institute of Psychiatry, Psychology and Neuroscience, King’s College London, UK; 2 South London and Maudsley NHS Foundation Trust, UK; 3 Department of Neuroimaging, Institute of Psychiatry, Psychology and Neuroscience, King’s College London, UK; 4 Department of Brain Sciences, Division of Psychiatry, Imperial College London, London, UK

**Keywords:** lithium, cognition, memory, processing speed, psychomotor speed, attention, verbal fluency, executive function

## Abstract

**Background:**

Lithium, a mainstay treatment for bipolar disorders, has shown promise in treating cognitive impairments. However, concerns about cognition-related side effects persist.

**Aims:**

We aimed to synthesise the evidence on how lithium affects cognition by comparing cognitive performance before and after starting lithium treatment.

**Methods:**

A systematic search was conducted to identify studies examining lithium’s effects on cognition. The review considered studies with adult human participants that reported quantitative cognitive outcomes using within-subject comparisons between lithium-absent and lithium-present conditions.

**Results:**

Thirty-two articles describing 30 studies were included (727 participants, approximately 54% female, mean age ± 50 years old). The studies exhibited significant heterogeneity within cognitive domains, including global cognition (15 studies), memory (19 studies), processing and psychomotor speed (8 studies), attention (9 studies), verbal fluency (4 studies) and executive function (6 studies). The included studies comprised 16 randomised controlled trials (RCTs) and 14 non-RCTs, with study populations ranging from individuals with affective disorders (13 studies) to neurocognitive disorders (11 studies) and healthy individuals (6 studies). Some studies reported cognitive enhancements, particularly in individuals with affective disorders, while others documented declines or mixed results.

**Conclusions:**

Definitive conclusions regarding lithium’s isolated cognitive effects remain elusive, particularly considering the influence of factors such as affective state, population and methodological heterogeneity among studies. Further research is needed to conclusively determine the raw cognitive impacts of lithium therapy, requiring larger RCTs across distinct populations. Prioritising the resolution of main symptoms should remain the primary therapeutic goal of lithium treatment.

## Background

Lithium, a cornerstone in the pharmacotherapy of bipolar disorders (BDs), manifests its therapeutic effects through pleiotropic neurobiological mechanisms. In practice, lithium’s efficacy extends to treating and preventing manic and depressive episodes, mitigating aggressive behaviours and anti-suicidal properties (Bauer and Gitlin, 2016a; Cipriani et al., 2013; Forlenza et al., 2014; Rybakowski, 2016; Vo et al., 2015). Beyond affective disorders, lithium also exhibits promise for its role in Alzheimer’s disease (AD) and mild cognitive impairment (MCI; Forlenza et al., 2011; Matsunaga et al., 2015).

The multifaceted neurobiological impact of lithium includes modulation of cell membrane properties, ion transport and distribution, neurotransmitter signalling and an increase in grey matter volume; thus, it is often perceived as being neuroprotective (Alda, 2015; Bauer and Gitlin, 2016b; Pasquali et al., 2010). It is stipulated that the neuroprotective effects emerge through the inhibition of glycogen synthase kinase-3 (GSK3), leading to enhanced cAMP response element-binding protein activity (Böer et al., 2008; Grimes and Jope, 2001). This, in turn, regulates brain-derived neurotrophic factor (BDNF) and anti-apoptotic B-cell lymphoma 2, fostering neuroprotection and neuronal survival (Chen and Chuang, 1999). Additionally, lithium’s modulation of the phosphoinositide cycle engages neuronal autophagy, impacting beta-amyloid and tau protein clearance linked to neurodegeneration (Berridge and Irvine, 1989; Forlenza et al., 2014; Zhang et al., 2011). In the context of clinical findings, this is corroborated by the increased cerebral grey matter volume and putatively reduced risk of dementia in bipolar patients treated with lithium (Hajek et al., 2014; Nunes et al., 2007).

Despite indications of neuroprotection, concerns persist regarding cognitive side effects and potential neurotoxicity after commencing lithium, as exemplified by conflicting evidence in the literature. Evidence suggests that cognitive impairment may be dose dependent, with higher lithium levels increasing the risk of adverse effects such as tremor and memory deficits, although the underlying mechanisms remain unclear (Ferensztajn-Rochowiak and Rybakowski, 2023). Additionally, older adults and individuals with pre-existing cognitive impairments may be more vulnerable due to higher serum lithium levels per administered dose (Grandjean and Aubry, 2009). Cognitive impairment is even listed as a side effect of long-term lithium treatment in some formularies (Joint Formulary Committee, 2024). In preclinical studies, lithium enhances synaptic plasticity, neurogenesis and neuroprotection by increasing long-term potentiation and synaptic density while also preventing neuronal apoptosis (Fiorentini et al., 2010; Yazlovitskaya et al., 2006). Lithium-treated rats exhibit better memory retention, rescued cognitive deficits via GSK-3β inhibition and showed cognitive-stabilising effects (King et al., 2014; Nocjar et al., 2007; Zhuo et al., 2022). A systematic review in 2008 explored lithium’s impact on cognition across human and animal studies; while the majority of investigations favoured neuroprotection, non-randomised human trials were more likely to report cognitive impairment or neurotoxicity (Fountoulakis et al., 2008). The most recent meta-analysis involving healthy individuals and affective disorder patients revealed a small but significant decline in verbal learning and memory associated with lithium, with no notable differences in short-term lithium use (Wingo et al., 2009). In BD, examining cognition reveals a paradox wherein lithium is also associated with cognitive impairment, particularly in domains such as verbal memory, processing and psychomotor speed, attention, and executive function (Green, 2006; Latalova et al., 2012). Irrespective of lithium treatment, meta-analyses consistently demonstrate a persistence of cognitive deficits across illness phases in BD (Robinson et al., 2006; Strakowski et al., 2004; Trotta et al., 2015). Notably, however, there is cognitive heterogeneity within this population, suggesting distinct cognitive profiles among BD patients (Tsapekos et al., 2020).

Lithium’s impact on cognition remains uncertain, marked by contrasting findings in past research constrained by methodological limitations. Unravelling the gaps in current studies unveils inconsistency in study designs, including variations in sample sizes, participant characteristics, treatment durations and outcome measure methods (Burdick et al., 2015; Pachet and Wisniewski, 2003). The predominant focus on BD patients reveals confounding variables within this population, such as psychiatric comorbidities, illness parameters and responses to lithium (e.g. excellent vs partial responders; Malhi et al., 2016; Rybakowski, 2015). Lithium’s cognitive impact in healthy individuals or those with neurodegenerative disorders has also not yet been systematically reviewed. Compared with previous reviews, which are over 15 years old, we aim to include a broader participant pool and consider various lithium treatment factors, such as doses and duration, using a within-subjects approach.

## Objectives

This systematic review aimed to synthesise the evidence surrounding lithium’s effects on human cognition in both healthy individuals and patients with neuropsychiatric disorders, from interventional studies. We planned to use within-subjects comparisons to examine whether lithium confers benefits or risks for cognition following initiation.

## Materials and methods

### Protocol and registration

This systematic review adhered to the established guidelines developed by the Cochrane Collaboration (Higgins et al., 2013) and Preferred Reporting Items for Systematic reviews and Meta-Analyses (PRISMA) guidelines (Page et al., 2021). We utilised Rayyan as a review and reference management software (Ouzzani et al., 2016). A protocol was developed and registered in PROSPERO (Registration number: CRD42023407053) prior to the searches being undertaken. Some amendments were deemed necessary following protocol registration (see Supplement 1).

### Study eligibility criteria

Studies were considered for inclusion if (i) the design was primary interventional research (randomised or non-randomised; controlled or uncontrolled); (ii) adult human participants were examined; (iii) a quantitative outcome pertaining to at least one objective, validated cognitive measure was reported; or (iv) a within-subjects comparison between lithium-present and lithium-absent conditions (pre-treatment vs on-treatment or on-treatment vs post-treatment) was reported. There were no language restrictions except if obtaining a suitable translation to English was not feasible. No restrictions were placed on doses and duration of lithium treatment or the participant population. No additional exclusion criteria were specified.

### Search strategy

A literature search was conducted using the OVID platform, encompassing the databases MEDLINE, EMBASE and PsycINFO. The final search was performed for studies up to 13th March 2023 using the strategy: (LITHIUM and (COGNIT* or NEUROCOGNIT* or MEMORY or EXECUTIVE or LEARNING or ATTENTION or PSYCHOMOTOR or PROCESSING SPEED or LANGUAGE) and (HUMAN or CLINICAL or PARTICIPANT or PATIENT or VOLUNTEER or PEOPLE)).ab,ot,ti. A manual search using reference lists and related articles in PubMed and Google Scholar was additionally performed.

Two reviewers (TNS, SM and SO in pairs) were blinded and independently evaluated the studies based on predefined eligibility criteria using Rayyan (Ouzzani et al., 2016). First, the reviewers screened the titles and abstracts of the retrieved articles. Subsequently, full-text versions of potentially eligible studies were obtained and examined in full. Any discrepancies were resolved through consensus between reviewers and, where indicated, through consulting a third reviewer (RS).

### Data extraction

Relevant data as pre-specified from the included studies were independently extracted by the two reviewers as above, using a standardised form consisted of the following: article information (author’s name, publication year), methodological information (study design, participants characteristics), lithium treatment information (preparation, doses, serum levels, duration) and outcome assessment (scores of cognitive measures respective to the cognitive domains). One reviewer (TNS) examined the discrepancies in the extracted data, and consensus was reached after a consultation with a third reviewer (RS). The same procedure was applied to assess the included studies’ risk of bias (RoB).

### RoB assessments

The RoB assessment was independently undertaken by the two reviewers as above, employing the Cochrane RoB tool for randomised controlled trials (RCTs) and the ROBINS-I tool for non-randomised controlled trials (non-RCTs) (Sterne et al., 2016, 2019). A tailored assessment was utilised to standardise the evaluation of both study designs, which encompassed the randomisation process, group comparison, potential confounders, deviations from interventions, missing outcome data, outcome measurement and selection of reported results.

### Outcomes

The primary outcome was cognitive performance. Cognitive assessments comprised objective tests, prioritising validated tests/batteries. Our definition of ‘cognition’ encompasses cognitive domains related to memory, processing and psychomotor speed, verbal fluency, attention and executive function. The measures were evaluated for their continuous value, such as score change over time after lithium intervention. We focused on within-subjects comparisons where participants act as their own control to reduce individual variability and illness parameters that can confound the results.

### Analysis

A systematic narrative synthesis was employed to describe the various intervention and outcome measures using the ‘Synthesis without meta-analysis’ approach outlined by Campbell et al. (2020). Within-subject comparisons were planned to describe the outcome relevant to intervention (pre-treatment, on-treatment or post-treatment). Where possible, subgroup analyses were planned to elucidate potential heterogeneity sources among study findings. When data sufficed, subgroup analyses were explored for variations in lithium doses, formulations, treatment durations, participant populations, study designs and cognitive domains. The results were presented through both summary and graphic tables. RoB level was considered received less weight in the interpretation of findings. This approach enabled the observation of patterns and trends within the data, in the absence of a formal meta-analysis.

## Results

### Study selection

**Figure 1.**
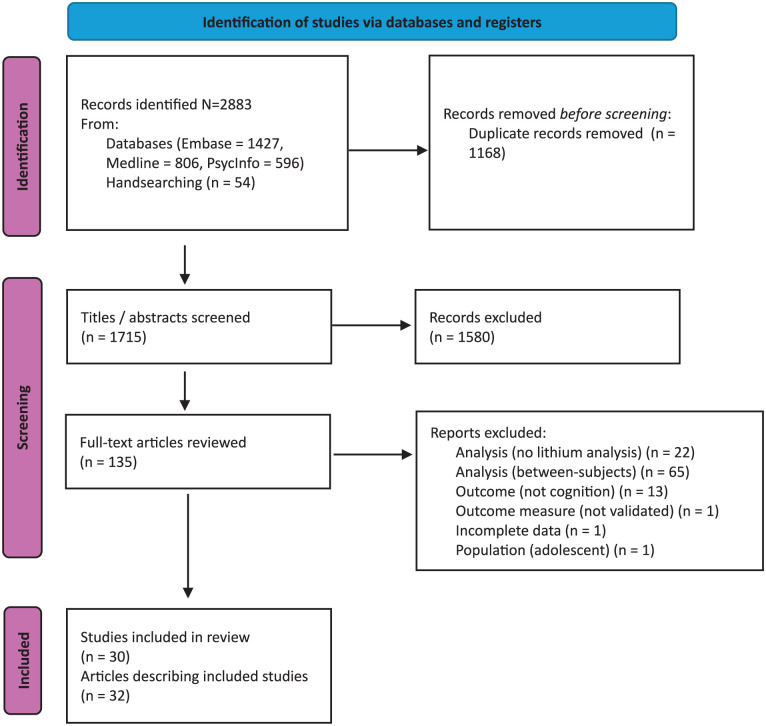
PRISMA 2020 flow diagram (Page et al., 2021) of the identified final studies.

The systematic search generated 1715 records via database and manual search after duplicates were removed. After the screening of titles and abstracts, 137 full-text articles underwent eligibility review. Ultimately, 32 articles describing 30 studies were included. The process of study identification is presented using the PRISMA flowchart (Figure 1; Page et al., 2021).

### Study characteristics

Table 1 presents the baseline characteristics of the 30 included studies, encompassing 16 RCTs and 14 non-RCTs (12 single-arm, 2 two-arm, 3 reversal and 1 discontinuation study). The reversal studies evaluated patients who were already on long-term lithium treatment; thus, a 2-week washout period was used to assess the off-lithium period before reinstatement measures; meanwhile, the discontinuation study assessed the long-term lithium value versus the off-lithium period (Christodoulou et al., 1981; Kocsis et al., 1993; Shaw et al., 1987; Telford and Worrall, 1978).

**Table 1.** Baseline study characteristics.

Authors	Year	Study design	Population	N	Mean Age(y)	% ♀	Intervention	Target daily dose/serum level	Mean dose/serum level	Duration (weeks)
Small *et al* .	1986	RCT	BD type 1 (Manic)	16/11*	38^*c^	27^*c^	Li	0.6 - 1.2 mmol/L	>= 0.6 mmol/L	8
Mardani *et al*,	2021	RCT	BD type 1 (Dep)	15	30	33	Li + VAL + CBZ	600 - 1500 mg BID	NR	1.5
Yucel *et al.*	2007	2 arm	BD type 1 & 2	12/6*	26^*b^	50^*b^	Li	0.5 - 0.7 mmol/L	0.7 mmol/L	208
Stoudemire A. *et al*,	1998	RCT	MDD (Dep)	17/4^a^	63^*c^	50^*c^	Li + ADx	150 mg BID titr. / 0.5 - 0.8 mEq/L	600 (245) mg / 0.55 (0.07) mEq/L	60
Kellner C.H. *et al.*	2016	RCT	MDD^b^	59/33*	70	63	Li	300 mg/day / 0.4 - 0.6 mEq/L	0.53 (0.27) mEq/L	24
Smith *et al.*	2010	RCT	MDD^b^	95/44*	58^*c^	73^*c^	Li + NTP	600 mg / 0.7 mEq/L	NR	24
Kocsis *et al.*	1993	1 arm^c^	Mixed affective	52/46*	54^*c^	61^*c^	Li	NR	0.75 (0.32) mEq/L	2^c^
Shaw E.D. *et al.*	1987	1 arm^c^	Mixed affective	28/22*	51^*c^	46^*c^	Li	NR	0.74 mol/L	2^c^
Christodoulou *et al.*	1981	1 arm^d^	Mixed affective	18/15*	47^*c^	80^*c^	Li	NR	1206 (205) mg / 0.92 (0.15 mEq/L)^e^	2.3^d^
Telford & Worrall	1978	1 arm^c^	Mixed affective	7^a^	40	86	Li + PHY	NR	0.83 (0.09) mmol/L	2^c^
Smigan & Perris	1983	1 arm	Mixed affective & SZA	53/36^f^	42^g *b^	57^*b^	Li sulphate	NR	0.63 mmol/L (0.16)	52
Zhuo *et al*.	2022	RCT	SZ	50	NR	NR	Li	0.4-0.8 mmol/L	NR	24
Small *et al.*	2003	RCT^h^	SZ & SZA	20/19*	21-53^i*b^	30^*b^	Li citrate^j^	300 mg BID / >= 0.5 mmol/L	0.60 mmol/L^k^	4
Devanand *et al.*	2022	RCT	AD	38	76	60	Li	150-600 mg / 0.2 - 0.6 mmol/L	263 (68) mg / 0.35 (0.14) mmol/L^k^	12
Nunes *et al.*	2013	RCT	AD	58/49*	77^*b^	58^*b^	Li GLU/CAR	300 mcg	300 mcg	65
Hampel *et al.*, Leyhe *et al.*	2009	RCT	AD	33/30*	68^*b^	54^*b^	Li sulphate	42 - 336 mg / 0.5 - 0.8 mmol/L	0.68 (0.23) mmol/L	10
Macdonald A. *et al.*	2008	2 arm	AD	22/8*	77^*c^	38^*c^	Li	100 mg / 0.3 - 0.8 mM	0.4 (0.05) mM	39.4 (mean)
Randels *et al.*	1984	1 arm	AD	10/6*	47-70^i*b^	0	Li + lecithin^l^	0.6 - 1 mEq/L	NR	4
Brinkman S.D. *et al.*	1982	1 arm	AD	10	53-81^i^	60	Li ion	300 mg BID	0.6 mEq/L	4
Forlenza *et al.*	2019, 2011	RCT	AMCI	31/27*	73 71^*b^	74^*b^	Li	150-600 mg BID / 0.25 - 0.5 mmol/L	0.39 mEq/L	104
Munoz-Moreno *et al.*	2017	RCT	HAND	11/6*	43^*b^	19^*b^	Li	400 mg BID / 0.4 - 0.8 mEQ/L	0.5 (0.3-0.8) mEq/L	48
Decloedt E.H. *et al.*	2016	RCT	HAND	32/30	39^*b^	82^*b^	Li	250 mg / 0.6 - 1.0 mmol/L	NR	24
Schifitto *et al.*	2009	1 arm	HAND (Dep)	15/13*	47^*b^	33^*b^	Li	300 mg BID / 0.16 - 0.8 mmol/L	0.42 mmol/L^g^	10
Sun *et al.*	2019	1 arm	Post-stroke	12/8*	71^*b^	50^*b^	Li	300-600 mg / 0.4 - 0.8 mmol/L	231 (150.5) mg / 0.275 (0.19) mM	8.5
Kohno *et al.*	2007	1 arm	Healthy	20	32	0	Li	600 mg / 0.6 mEq/L	0.78 (0.11) mEq/L	4
Bell *et al.*	2005	RCT	Healthy	9	26	33	Li	900 mg	0.39 - 0.77 mmol/L	2
Stip *et al.*	2000	RCT	Healthy	15/13*	32^*c^	46^*c^	Li	1050-1950 mg / 0.8 mmol/L	1569 mg	3
Calil *et al.*	1990	RCT^c^	Healthy	20/17	23^*c^	35^*c^	Li	300-600 mg BID / 0.6 - 1 mEq/L	1252 (265) mg / 0.8 (0.1) mEq/L	4
Kropf *et al.*	1979	RCT	Healthy	12	20-30^i^	0	Li sulphate	24-36 mval	0.54 (0.15) mmol/L	2
Müller-Oerlinghausen *et al.*	1977	2 arm	Healthy	10	NR	70	Li sulphate	36 mval / 1 mmol/L	1.1 (0.25) mmol/L	1

Abbreviations: N = number of participants, y = years,♀ = female, RCT = randomised controlled trial, AD = Alzheimer’s Disease, Li = lithium carbonate, mg = milligrams, mmol/L = millimoles per litre, BD = bipolar disorder, Dep = depressed, VAL = sodium valproate, CBZ = carbamazepine, BID =  twice daily, NR = not reported, AMCI = amnesic mild cognitive impairment, mEq = milliequivalents, nRCT = non-randomised controlled trial, mM = millimolar, HAND = HIV-associated neurocognitive disorders, MDD = major depressive disorder, GLU = gluconate, CAR = carbonate, mcg = microgram, NTP = nortriptyline, ADx = antidepressant, titr. = titrated, SDAT = senile dementia of the Alzheimer’s type, PHY = physostigmine, mval = millivalents.

* = N for baseline/ N completers, ^*c^ = from N completers, ^*b^ = from N baseline, ^a^ = number of li tolerant, ^b^ = 20-52% participants established psychotic features, ^c^ = reversal li study (on-off-off-on-on; two weeks off before two weeks re-initiation), ^d^ = lithium discontinuation, ^e^ = prior to washout, ^f^ = variability of N 34-35 on a few assessments, ^g^ = median value, ^h^ = double-blind crossover RCT, ^i^ = age range, ^j^ = patients on clozapine, ^k^ = mean value in li responders, ^l^ = introduced in last 2 weeks

Predominantly, included studies introduced lithium as an intervention in conjunction with standard care. Notably, five studies initiated lithium concurrently with other agents, such as anticonvulsants, antidepressants, physostigmine and lecithin (Mardani et al., 2021; Randels et al., 1984; Smith et al., 2010; Stoudemire et al., 1998; Telford and Worrall, 1978). Study populations included BDs (3 studies), major depressive disorders (3 studies), mixed affective disorders (a mix of participants with unipolar and BDs, with/without psychosis; 5 studies), schizophrenia and schizoaffective disorders (2 studies), AD (6 studies), amnestic MCI (1 study), HIV-associated neurocognitive disorders (HANDs; 3 studies), post-stroke (1 study) and healthy volunteers (6 studies). Four studies addressed non-euthymic states (depressed or manic) at baseline (Mardani et al., 2021; Schifitto et al., 2009; Small et al., 1986; Stoudemire et al., 1998). The mean sample size at baseline was *N* = 24 (SD = 16, range 4–59). We categorised these populations into three main groups: BDs, schizophrenia and other affective disorders (including major depressive disorders, mixed affective disorders and schizoaffective disorders), neurocognitive disorders (those with AD, amnestic MCI and HANDs) and healthy participants. This allows for a clearer understanding of lithium’s effects across different diagnostic groups.

The preparation most frequently used was lithium carbonate, while other formulations included lithium sulphate, lithium gluconate, lithium citrate and lithium ion (Brinkman et al., 1982; Hampel et al., 2009; Kropf and Müller-Oerlinghausen, 1979; Leyhe et al., 2009; Müller-Oerlinghausen et al., 1977; Nunes et al., 2013; Small et al., 2003; Smigan and Perris, 1983). Target doses varied from 300 μg to 1950 mg per day, and target serum levels ranged from 0.2 to 1.0 mmol/L. When reported, actual mean doses varied from 300 μg to 1569 mg, and mean serum levels ranged from 0.27 to 0.96 mmol/L. The overall treatment duration (defined as the longest period of lithium exposure after initiation; in reversal studies, lithium reinstatement after the washout period; in discontinuation study, off-lithium period) ranged from 1 to 208 weeks. Due to the heterogeneity of the study designs and outcome reporting variability, it was not deemed to be suitable to conduct a meta-analysis. Therefore, a narrative description of the results is presented.

### Cognitive domains

#### Global cognition

Fifteen studies examined the impact of lithium on global cognitive function (Table 2). Five studies reported significant improvements (Decloedt et al., 2016; Small et al., 1986; Stoudemire et al., 1998; Telford and Worrall, 1978; Zhuo et al., 2022), three noted no significant changes (Schifitto et al., 2009; Sun et al., 2019; Munoz-Moreno et al., 2017), and seven showed mixed results (Devanand et al., 2022; Forlenza et al., 2011, 2019; Hampel et al., 2009; Leyhe et al., 2009; Kellner et al., 2016; Macdonald et al., 2008; Nunes et al., 2013; Randels et al., 1984). In BDs, studies involving bipolar patients showed improvements in global cognition, particularly when lithium was used following a manic episode. For example, one study reported improved scores on the Wechsler Adult Intelligence Scale (WAIS) Performance IQ and Full Scale IQ after 8 weeks of lithium treatment (Small et al., 1986). In other affective disorders and schizophrenia patients, some studies demonstrated improvements in global cognition. A 60-week study involving lithium-responsive depressed individuals showed increased scores on the Mattis Dementia Rating Scale (Stoudemire et al., 1998). Additionally, a 2-week investigation involving additional physostigmine injection reported improvement within the same WAIS subtests as in the bipolar study mentioned above (Telford and Worrall, 1978). Another study involving schizophrenia patients showed an improvement in the Global Deficit score after 24 weeks of lithium treatment (Zhuo et al., 2022). In neurocognitive disorders, a study in HANDs patients also reported improvements in global cognition (Decloedt et al., 2016).

**Table 2.** Lithium effects on global cognition.

**Study**	**Year**	**Population**	**N**	**Duration (weeks)**	**Average serum level (mmol/L)**	**Cognitive measures**	**Score Pre-Li (mean, SD)**	**Score Post-Li (mean, SD)**	**Significance**
							[score change over time (mean, SD)]		
**Small *et al.***	1986	BD type 1 (Manic)	16/11*	8	0.6	WAIS Perf. IQ WAIS Full IQ	84.9 86.5	93.8 95.4	↑, p = 0.029 ↑, p = 0.031
**Kellner *et al.***	2016	MDD	59/33*	24	0.5	MMSE	27.4 (2.3)	27.8 (2.4)	↑, NR
**Stoudemire *et al.***	1998	MDD (Dep)	4	60	0.6	MDRS	130 (10.58)	137.25 (6.9)	↑, p = 0.04
**Telford *et al.***	1978	Mixed affective	7	2	0.8	WAIS Perf. IQ WAIS Full IQ	111.43 (13.49) 115.14 (11.02)	129.43 (9) 125.43 (7.76)	↑, p < 0.05 ↑, p < 0.05
**Zhuo *et al.***	2022	SZ	50	24	0.4	GDS	3.2 (2.14)	2.77 (1.26)	↑, p < 0.05
**Devanand *et al.***	2022	AD	38	12	0.4^y^	MMSE SIB	0.9 (-.03 to 2.2)^a^ 2.1 (-1.1 to 5.4)^a^		↑, NR ↑, NR
**Nunes *et al.***	2013	AD	58/49*	65	300 mcg^x^	MMSE	19.48 (0.67)	19.82 (0.9)	NC, NR
**Hampel *et al.*, Leyhe *et al.***	2009	AD	33/30*	10	0.7	MMSE ADAS-Cog	23.6 (1.6) 15.8 (4.2)	22.6 (3.5) 15.6 (4.4)	NC, NS
**Macdonald *et al.***	2008	AD	22/8*	39.4	0.4	MMSE	18.5 (4.3)	4.8 (5.5)^a^	↑, SNC
**Randels *et al.***	1984	AD	10/6*	4	0.6 – 1^x^	MMSE	0-14^b^	0-14^b^	NC, NR
**Forlenza *et al.***	2011, 2019	AMCI	31/27*	104	0.4	CDR-SoB ADAS-Cog	−0.22 (0.9)^a^ 0.83 (1.1)^a^		↑, NR ↑, NR
**Munoz-Moreno *et al.***	2017	HAND	11/6*	48	0.5 (0.3-0.8)^c^	NPZ-7	-0.50 (0.40)	-0.26 (0.21)	↑, NS
**Decloedt *et al.***	2016	HAND	32	24	0.6 - 1.0	GDS	1.08 (0.83–1.44)^c^	0.73 (0.35–0.92)^c^	↑, p < 0.0001
**Schifitto *et al.***	2009	HAND	15/13*	10	0.4	NPZ	0.62 (3.15)	2.32 (3.38)	↑, NS
**Sun *et al.***	2019	Post-stroke	12/8*	8.5	0.3	MMSE MoCA	28 (25-29)^c^ 23 (16–24)^c^	NR 21 (16–24)^c^	NR, NS ↓, NS

Abbreviations: N = number of participants, mmol/L = millimoles per litre, Li = lithium, BD = bipolar disorder, AD = Alzheimer’s Disease, MMSE = Mini Mental State Examination, SIB = Severe Impairment Battery, SZ = schizophrenia, GDS = Global Deficit/Disability Score, NR = not reported, NS = not significant, MoCA = Montreal Cognitive Assessment, AMCI = amnestic mild cognitive impairment, BID = two times per day, CDR-SoB = Clinical Dementia Rating, ADAS-Cog = Alzheimer’s Disease Assessment Scale-Cognitive Subscale, HAND = HIV-associated Neurocognitive Disorders, mcg = microgram, NC = no/negligible change, mEq = milliequivalents, NPZ = NeuroPsychological Zero, SNC = significance not clear, MDD = major depressive disorder, MDRS = Mattis Dementia Rating Scale, WAIS = Weschler Adult Intelligence Scale, Perf = Performance, ↑ = improved, ↓ = declined.

* = N baseline / N completers, ^a^ = mean change, ^b^ = range, ^c^ = median, interquartile range, ^y^ = mean value in li responders, ^x^ = where actual mean serum not available, target serum level or dose is reported.

#### Memory

Nineteen studies evaluated the impact of lithium on memory (Table 3). Six studies reported significant improvements (Bell et al., 2005; Calil et al., 1990; Christodoulou et al., 1981; Smigan et al., 1983; Stip et al., 2000; Yucel et al., 2007), three noted significant declines (Kocsis et al., 1993; Kropf and Muller-Oerlinghausen, 1979; Shaw et al., 1987), and 10 showed mixed or no significant changes (Brinkman et al., 1982; Decloedt et al., 2016; Forlenza et al., 2011, 2019; Kohno et al., 2007; Munoz-Moreno et al., 2017; Schifitto et al., 2009; Small et al., 1986, 2003; Smith et al., 2010; Sun et al., 2019). In BDs, a 4-year investigation among euthymic patients with bipolar type 1 and 2 showed improved verbal memory using the California Verbal Learning Test (Yucel et al., 2007), whereas Small et al. (1986) found no changes in manic BD type 1 patients. A year-long study of individuals with mixed affective and schizoaffective disorders demonstrated improvements in immediate and delayed recall tasks (Smigan and Perris, 1983), while two reversal studies involving lithium discontinuation and reinitiation for 2-week periods in mixed affective disorder patients reported a decline in Buschke Selective Reminding Test scores (Kocsis et al., 1993; Shaw et al., 1987). Additionally, one study indicated memory improvements post-discontinuation of lithium for 16 days in patients with recurrent affective illness (Christodoulou et al., 1981). Among neurocognitive disorders, a 104-week RCT in amnestic MCI suggested memory improvements without clear within-subjects significance (Forlenza et al., 2019); however, studies in AD and post-stroke cohorts reported no significant changes (Brinkman et al., 1982; Sun et al., 2019). In healthy volunteers, three short-term (2–4 weeks) studies showed improved responses in verbal and working memory tasks (Bell et al., 2005; Calil et al., 1990; Stip et al., 2000), while another short-term study showed worsened free recall and recognition time of learned nouns (Kropf and Müller-Oerlinghausen, 1979).

**Table 3.** Lithium effects on memory outcomes.

**Authors**	**Year**	**Population**	**N**	**Duration (weeks)**	**Average serum level (mmol/L)**	**Cognitive measures**	**Score Pre-Li (mean, SD)**	**Score Post-Li (mean, SD)**	**Significance**
							[score change over time (mean, SD)]		
**Small *et al.***	1986	BD type 1 (Manic)	11	8	> 0.6	WAIS DS	7.6	8.5	↑, NS
**Yucel *et al.***	2007	BD type 1 & 2	12/6*	208	0.7	CVLT	56	64	↑, p < 0.05
**Smith *et al.***	2010	MDD	44	12^>^	0.7^x^	R-AVLT % ret RMT Paired-words RMT Short-story RCFT % ret AMI	40.1 (2.9) 4.0 (0.2) 70.0 (6.1) 40.9 (2.2) 33.6 (1.0)	22.0 (3.7)^a^ -0.5 (0.2)^a^ 17.5 (8.0)^a^ 6.8 (3.1)^a^ 1.5 (1.2)^a^	↑, SNC^^^ ↓, SNC^^^ ↑, SNC^^^ ↑, SNC^^^ ↑, SNC^^^
**Kocsis *et al.***	1993	Mixed affective	46	2^#^	0.8	BSRT SCLTR BSRT SLTR BSRT CLTR BSRT LTR BSRT R BSRT S R	1.3 (1.15) 1.37 (0.98) 8.5 (3.89) 10.22 (4.02) 11.98 (2.97) 1.18 (0.70)	0.93 (0.90) 1.11 (0.81) 6.70 (4.27) 8.98 (4.22) 11.07 (3.23) 0.93 (0.50)	↓, p < 0.05 ↓, p < 0.05 ↓, p < 0.05 ↓, p < 0.05 ↓, p < 0.05 ↓, p < 0.05
**Shaw *et al.***	1987	Mixed affective	22	2^#^	0.7	BSRT IR BSRT STR BSRT LTS BSRT LTR BSRT SLTR BSRT CLTR BSRT SCLTR BSRT R BSRT S R	6.09 (2.09) 1.45 (1.37) 11.91 (3.28) 12.04 (3.20) 1.77 (1.11) 9.86 (3.31) 1.77 (1.4) 13.32 (2.36) 1.07 (0.76)	5.86 (2.03) 1.86 (1.49) 10.23 (4.20) 10.18 (4.18) 1.38 (0.95) 7.14 (41.31) 1.18 (1.09) 11.91 (3.49) 0.72 (0.39)	↓, NS ↑, NS ↓, p < 0.01 ↓, p < 0.005 ↓, p < 0.01 ↓, p < 0.001 ↓, p < 0.005 ↓, p < 0.005 ↓, p < 0.001
**Christodoulou *et al.***	1981	Mixed affective	15	2.3⁺	0.9	WAIS Paired Words, 1st WAIS Paired Words, 2nd WAIS Paired Words, 3rd R-AVLT, 1st R-AVLT, 2nd R-AVLT, 3rd BVRT Imm. Rep (errors) BVRT Del. Rep (errors) WAIS DS Fwd WAIS DS Bwd	3.93 (2.42) 5.96 (2.48) 7.6 (2.28) 4.18 (2.12) 5.93 (2.67) 7.96 (3.43) 9.29 (4.01) 10.08 (4.92) 5.47 (1.18) 3.70 (1.08)	4.21 (1.84) 6 (2) 7.5 (2.6) 4.14 (2.03) 7.35 (1.49) 9.07 (2.64) 5.00 (3.30) 5.54 (3.17) 5.43 (1.11) 4.16 (1.45)	↑, NS NC, NS NC, NS NC, NS ↑, p < 0.01 ↑, NS ↑, p < 0.005 ↑, p < 0.02 NC, NS ↑, NS
**Smigan *et al.***	1983	Mixed affective & SZA	53/36*	48	0.6	30 Fig Imm 30 Word-Pair Imm 30 Person-Data Imm 30 Face Imm 30 Figure Del 30 Word-Pair Del 30 Person-Data Del 30 Face Del	23.8 (7)^b^ 20 (10)^b^ 13.2 (8)^b^ 14.1 (9)^b^ 21.9 (8)^b^ 13.6 (10)^b^ 12.1 (9)^b^ 12.5 (10)^b^	25.2 (8)^b^ 17.7 (8)^b^ 15.1 (11)^b^ 18.5 (9)^b^ 23.2 (8)^b^ 14.7 (11)^b^ 12.6 (10)^b^ 15.5 (12)^b^	↑, NS ↓, NS ↑, NS ↑, p < 0.001 ↑, NS ↑, NS ↑, NS ↑, p < 0.02
**Small *et al.***	2003	SZ, SZA	20/19	4	SZ: 0.5, SZA: 0.6	Imm R Visual Recognition	SZ: 5, SZA: 8.8 SZ: 8.4, SZA: 12.3	SZ: 3.8, SZA: 7.2 SZ: 7, SZA: 12.6	SZ: ↑ SZA: ↑, NR SZ: ↓ SZA: ↑, NR
**Forlenza *et al.***	2011, 2019	AMCI	31/27*	104	0.4	CERAD Del R CERAD Fig R	0.16 (0.4) 0.17 (0.3)		↑, NR ↑, NR
**Munoz-Moreno *et al.***	2017	HAND	11/6*	48	0.5 (0.3-0.8)	WAIS DS Fwd WAIS DS Bwd WAIS Letters-number CVLT-II Total A list (CVLT-II)	0.11 (0.68)^a^ –0.01 (0.69)^a^ 0.01 (0.91)^a^ 0.33 (0.60)^a^ 0.61 (0.88)^a^		↑, NR NC, NR NC, NR ↑, NR ↑, NR
**Decloedt *et al.***	2016	HAND	32	24	0.6 - 1.0^x^	HVLT-R RCFT copy RCFT 3 min	5.0 (5.0–6.0)^b^ 21.70 (7.87) 10.44 (4.59)	7.0 (7.0–8.0)^b^ 21.70 (7.87) 10.44 (4.59)	↑, NR NC, NR NC, NR
**Schifitto *et al.***	2009	HAND	15/13*	10	0.4^b^	R-AVLT Total R-AVLT Trial 5 R-AVLT R Inference R-AVLT Del R	32.47 (6.01) 8.33 (2.26) 6.33 (2.02) 5.73 (2.25)	37.14 (6.89) 9.64 (2.17) 5.93 (1.69) 5.54 (1.81)	↑, NS ↑, NS ↓, NS ↓, NS
**Brinkman *et al.***	1982	SDAT	10	4	0.6	BSRT LTR BSRT LTS BSRT R	17.9 (7.9) 25.1 (9.7) 40.9 (10.5)	15.7 (8.4) 20.0 (10.3) 40 (10.5)	↓, NS ↓, NS ↓, NS
**Sun *et al.***	2019	Post-stroke	12/8*	8.5	0.3	HVLT-R	4.8 (3.4)	6.3 (2.7)	↑, NS
**Kohno *et al.***	2007	Healthy	20	4	0.8	AVLT Imm AVLT Recent	5.9 (1.8) 8.4 (1.8)	6.5 (1.7) 8.7 (1.7)	↑, NS ↑, NS
**Bell *et al.***	2005	Healthy	9	2	0.39 - 0.77^x^	Arrow Condition (ms) DS Fwd (ms)	454.24 (17.32) 913.18 (62.13)	422.81 (22.73) 818.00 (53.79)	↑, p = 0.017 ↑, p = 0.028
**Stip *et al.***	2000	Healthy	13	3	0.8^x^	Auditory DS CVLT Explicit CVLT Implicit	NR NR NR	NR NR NR	↑, p < 0.03 NS NS
**Calil *et al.***	1990	Healthy	17	4	0.8	WAIS Memory Span R-AVLT	10.7 (1.8) 9.1 (1.5)	11.8 (1) 10.3 (1.4)	↑, p < 0.04 ↑, p < 0.03
**Kropf *et al.***	1979	Healthy	12	2	0.5	Learning Learning Time Free R Free R Time Recognition Recognition Time R + Motivation R Time + Motivation R – Motivation R Time - Motivation	10.75 (4.07) 429 (234) 12.75 (1.86) 83.9 (60.82) 15.08 (2.02) 38.1 (11.4) 12.5 (2.43) 112 (68.5) 13 (1.26) 55.8 (22.7)	11.91 (4.9) 523.4 (254) 7.83 (5.3) 78.9 (51.3) 15.08 (0.66) 77.75 (56) 13 (1.67) 154 (92.3) 13.5 (2.16) 50 (15.8)	↓, NS ↓, NS ↓, p = 0.005 ↑, NS NC, NS ↓, p = 0.02 ↑, NR ↓, NR ↑, NR ↑, NR

Abbreviations: N = number of participants, mmol/L = millimoles per litre, Li = lithium, HVLT-R = Hopkins Verbal Learning Test – Recall, NS = not significant, NR = not reported, HAND = HIV-associated Neurocognitive Disorders, WAIS = Wechsler Adult Intelligence Scale, DS = Digit Span, Fwd = Forward, Bwd = Backward, NC = no/negligible change, CVLT = California Verbal Learning Test, RCFT = Rey Complex Figure Test, AMCI = amnestic mild cognitive impairment, CERAD = Consortium to Establish a Registry for Alzheimer’s Disease, Del = Delayed, Fig = Figure, R = Recall, Imm = Immediate, MDD = major depressive disorder, R-AVLT= Rey Auditory Verbal Learning Test, ret = retention, SNC = significance not clear, SZ = schizophrenia, SZA = schizoaffective, RMT = Randt Memory Test, AMI = Autobiographical Memory Interview, BD = bipolar disorder, ms = milliseconds, BSRT = Buschke Selective Reminding Test, IR = Immediate Recall, STR = Short Term Recall, LTS = Long Term Storage, LTR = Long Term Retrieval, SLTR = Slope of LTR, CLTR = Consistent LTR, SCLTR =Slope of CLTR, S R = Slope of Total Recall, SDAT = senile dementia of Alzheimer’s type, BVRT = Benton Visual Retention Test, Imm. Rep = Immediate Reproduction, Del. Rep = Delayed Reproduction, ↑ = improved, ↑ = declined.

* = N baseline / N analysis, ^a^ = mean change, ^b^ = median value, with/without interquartile range, ^>^ = actual study duration was longer (24 weeks) but cognition only reported after 12 weeks, ^x^ = where actual mean serum not available, target serum level or dose is reported, ^#^ = long-term lithium reversal study (after 2 weeks washout as ‘pre-li’ value, and after 2 weeks re-initiation as ‘post-li’ value), ⁺ = post-lithium discontinuation, ^^^ = significant results at the end of 12 weeks period.

#### Processing and psychomotor speed

Eight studies investigated the impact of lithium on processing and psychomotor speed (Table 4). Two studies reported significant improvements (Calil et al., 1990; Small et al., 1986), one noted significant declines (Shaw et al., 1987) and five showed mixed or no significant changes (Decloedt et al., 2016; Kocsis et al., 1993 Kohno et al., 2007; Munoz-Moreno et al., 2017; Schiffito et al., 2009). An 8-week study in manic bipolar patients reported significant improvements in WAIS Digit Symbol and Block Design tasks (*p* < 0.05; Small et al., 1986). Two reversal studies on mixed affective disorder patients showed varying outcomes in finger tapping tests upon lithium reinitiation: one study indicated no significant difference, while the other demonstrated a significant decrease in performance (Kocsis et al., 1993; Shaw et al., 1987). Among all HAND populations, two studies did not clearly document the significance of within-subjects analyses, one predominantly reporting worsened outcomes and another showing mixed results (Decloedt et al., 2016; Munoz-Moreno et al., 2017); while a third study reported negligible or non-significant improvements (Schifitto et al., 2009). Healthy volunteers exhibited improved WAIS Digit Symbol scores after 4 weeks (Calil et al., 1990) but no changes in other tests such as Trail Making Task (TMT-A; Kohno et al., 2007).

**Table 4.** Lithium effects on processing and psychomotor speed.

**Authors**	**Year**	**Population**	**N**	**Duration (weeks)**	**Serum level (mmol/L)**	**Cognitive measures**	**Score Pre-Li (mean, SD)**	**Score Post-Li (mean, SD)**	**Significance**
							[score change over time (mean, SD)]		
**Small *et al.***	1986	BD type 1	11	8	0.6 - 1.2^x^	WAIS Picture Arrangement WAIS Object Assembly WAIS Block Design WAIS DSym	6.3 6.7 6.8 6.6	7.8 7.3 8.8 7.2	↑, NS ↑, NS ↑, p = 0.025 ↑, p = 0.047
**Kocsis *et al.***	1993	Mixed affective	46	2^#^	0.8	FT, left FT, right	45.97 (10.82) 51.47 (11.63)	44.95 (11.30) 50.49 (11.55)	↓, NS ↓, NS
**Shaw *et al.***	1987	Mixed affective	22	2^#^	0.74	Combined FT	54.56 (5.6)	52.23 (6.64)	↓, p < 0.02
**Munoz-Moreno *et al.***	2017	HAND	11/6*	48	0.5 (0.3 – 0.8)^b^	TMT-A SDMT GPT Dom GPT Non-dom	0.90 (1.56)^a^ 0.25 (0.24)^a^ -0.08 (0.71)^a^ -0.16 (0.39)^a^		↑, NR ↑, NR ↓, NR ↓, NR
**Decloedt *et al.***	2016	HAND	32	24	0.6 - 1.0^x^	FT Non-dom GPT Non-dom TMT-A Color Trails 1 WAIS DSym	7.58 (6.70–8.73)^b^ 96.60 (79.96–123.04)^b^ 59.54 (46.46–88.58)^b^ 58.84 (50.58–82.15)^b^ 28.84 (10.55)	6.78 (6.30–7.88)^b^ 80.36 (75.16–96.79)^b^ 47.05 (36.71–55.63)^b^ 64.23 (47.39–76.13)^b^ 29.24 (11.28)	↓, NR ↓, NR ↓, NR ↓, NR ↑, NR
**Schifitto *et al.***	2009	HAND	15/13*	10	0.4^b^	WAIS DSym GPT Dom (s) GPT Non-dom (s) Timed Gait (s)	39.27 (9.50) 77.07 (7.90) 83.07 (12.02) 8.64 (0.79)	40.00 (8.99) 73.57 (12.57) 80.64 (13.99) 8.05 (0.74)	↑, NS ↑, NS ↑, NS ↑, NS
**Kohno *et al.***	2007	Healthy	20	4	0.8	TMT-A	63 (16.6)	63.5 (20.4)	↓, NS
**Calil *et al.***	1990	Healthy	17	4	0.8	ETEP RT Visual ETEP RT Auditory WAIS DSym	211 (38) 208 (31) 62.3 (8.7)	198- (27) 206- (18) 66.3 (8.6)	↑, NS ↑, NS ↑, p < 0.01

Abbreviations: N = number of participants, mmol/L = millimoles per litre, Li = lithium, BD = bipolar disorder, HAND = HIV-associated Neurocognitive Disorders, TMT-A = Trail Making Test A, NR = not reported, SDMT = Symbol Digit Modalities Test, GPT = Grooved Pegboard Test, Dom = dominant hand, Non-dom = non-dominant hand, FT = Finger Tapping, WAIS = Weschler Adult Intelligence Scale, DSym = Digit Symbol, s = seconds, NS = not significant, ETEP RT = Effort Test for Evaluating Perceptual and Executive Abilities - Reaction Time, ↑ = improved, ↓ = declined.

* = N baseline / N analysis, ^a^ = mean change, ^b^ = median value, with/without interquartile range, - = negative values indicate improvement, ^x^ = where actual mean serum not available, target serum level is reported, ^#^ = long-term lithium reversal study (after 2 weeks washout as ‘pre-li’ value, and after 2 weeks re-initiation as ‘post-li’ value).

#### Attention

Nine studies evaluated the impact of lithium on attention (Table 5). Two studies reported significant improvements (Small et al., 1986, 2003), two noted declines (Schiffito et al., 2009; Müller-Oerlinghausen et al., 1977) and five showed mixed or no significant changes (Bell et al., 2005; Decloedt et al., 2016; Forlenza et al., 2011, 2019; Kohno et al., 2007; Stip et al., 2000). A study focusing on manic bipolar patients showed a significant increase in WAIS subtests, such as Arithmetic and Picture Completion (Small et al., 1986). Similarly, another one involving individuals with schizophrenia and schizoaffective disorders reported a significant improvement in the Letter Number Length test during 4 weeks of lithium treatment (Small et al., 2003). A long-term trial in amnestic MCI reported mixed outcomes unclear within-subjects significance (Forlenza et al., 2011, 2019). In HANDs studies, one exhibited significantly slower reactions on one of two attention tasks (Schiffito et al., 2009), while the other reported an improvement in attention without a clear significance (Decloedt et al., 2016). Among healthy volunteers, a high-dose trial (1050–1950 mg) reported declines in attention and concentration tasks (Müller-Oerlinghausen et al., 1977), while others found no significant changes (Bell et al., 2005; Kohno et al., 2007).

**Table 5.** Lithium effects on attention.

Authors	Year	Population	N	Duration (weeks)	Average serum level (mmol/L)	Cognitive measures	Score Pre-Li (mean, SD)	Score Post-Li (mean, SD)	Significance
							[score change over time (mean, SD)]		
Small *et al.*	1986	BD type 1	11	8	0.6 - 1.2^x^	WAIS Arithmetic WAIS Pic. Completion	7.7 7.4	9.6 9.5	↑, p = 0.038 ↑, p = 0.019
Small *et al.*	2003	SZ & SZA	20/19*	4	0.6^y^	Letter Number Length	3.3	3.9	↑, p < 0.05
Forlenza *et al.*	2011, 2019	AMCI	31/27*	104	0.4	SLN TMT A	−0.67 (0.5)^a^ −0.80 (6.4)^a^-		↓, NR ↑, NR
Decloedt *et al.*	2016	HAND	32	24	0.6 - 1.0^y^	WAIS DS PASAT	6.06 (1.27) 15.0 (12.0–19.5)^b^	6.34 (1.50) 17.50 (11.5–23.0)^b^	↑, NR ↑, NR
Schifitto *et al.*	2009	HAND	15/13*	10	0.4^b^	CalCAP Choice CalCAP Sequential	421.00 (38.20) 558.80 (150.77)	443.07 (48.81)- 587.86 (162.80)-	↓, p = 0.033 ↓, NS
Kohno *et al.*	2007	Healthy	20	4	0.8	CPT	348 (36)	365 (71)	↓, NS
Bell *et al.*	2005	Healthy	9	2	0.39 - 0.77^x^	Attended trial (ms) Unattended trial (ms)	302.51 (15.54) 313.68 (19.20)	291.42 (19.08) 321.39 (18.44)	↑, NS ↓, NS
Stip *et al.*	2000	Healthy	13	3	0.8^x^	RT target^c^ Visuomotor pursuit task^d^ RT distracters^e^ RT distracters^f^	NR NR NR NR	NR NR NR NR	↓, p NR NS NR, p < 0.03 ↓, SNC
Müller-Oerlinghausen *et al.*	1977	Healthy	10	1	36 mval^x^	D2 KVT (sec) KVT (errors)	NR 544 13.3	4^g^ 611- 10.8	↑, SNC ↓, p < 0.05 ↑, NR

Abbreviations: N = number of participants, mmol/L = millimoles per litre, Li = lithium, BD = bipolar disorder, HAND = HIV-associated Neurocognitive Disorders, WAIS = Weschler Adult Intelligence Score, DS = Digit Span, NR = not reported, PASAT = Paced Auditory Serial Addition Test, AMCI = amnestic mild cognitive impairment, SLN = Sequence of Letters and Numbers, TMT A = Trail Making Test A, CalCAP = California Computerized Assessment Package reaction time test, NS = not significant, CPT = Continuous Performance Test, SZ = schizophrenia, SZA = schizoaffective, RT = reaction time, Pic. = Picture, mval = millivalent, D2 = D2 Test of Attention, SNC = significance not clear, KVT = Konzentrations-Verlaufs-Test or Concentration Performance Test, sec = seconds, ↑ = improved, ↓ = declined.

* = N baseline / N analysis, ^a^ = mean change, - = negative values indicate improvement, ^b^ = median value, with/without interquartile range, ^c^ = for a target, ^d^ = moving-target visuomotor pursuit task with a digit span recall, ^e^ = for a target amongst distracters simultaneously, ^f^ = for a target amongst distracters one at a time, ^g^ = percent increased, ^x^ = where actual mean serum not available, target serum level or dose is reported, ^y^ = mean value in li responders.

#### Executive function

Six studies evaluated the impact of lithium on executive functions (four of which examined verbal fluency specifically and are thus reported separately below); see Table 6. No study reported significant improvements or declines. In bipolar type 1 patients, one study in euthymic patients focused on inhibition control and one in manic patients focused on the domains of Information, Vocabulary, and Verbal IQ reported non-significant changes (Mardani et al., 2021; Small et al., 1986). Of three neurocognitive disorder studies where the significance was unclear, two reported an overall worsening (Forlenza et al., 2019; Munoz-Moreno et al., 2017) and one reported an overall improvement (Decloedt et al., 2016) in executive function. One study in healthy volunteers showed no significant changes using the Trail Making Test B (Kohno et al., 2007).

**Table 6.** Lithium effects on verbal fluency and executive function.

Authors	Year	Population	N	Duration (weeks)	Average serum level (mmol/L)	Cognitive measures	Score Pre-Li (mean, SD)	Score Post-Li (mean, SD)	Significance
							[score change over time (mean, SD)]		
Mardani *et al.*	2021	BD type 1	15	1.5	600 - 1500 mg^x^	Go/No-go Comm. error Go/No-go Om. error Average RT (ms)	1.86 (2.58) 0.33 (0.72) 369.46 (46.06)	2.46 (3.46)^+^ 3.06 (8.09)^+^ 387.33 (78.60)^+^	↓, NS ↓, NS, ↓, NS
Small *et al.*	1986	BD type 1	11	8	0.6^y^	*WAIS Verbal IQ*	*88.7*	*96.9*	*↑, NS*
Forlenza *et al.*	2011, 2019	AMCI	31/27*	104	0.4	TMT B	1.95 (8.6)^a^^+^		↓, NR
Munoz-Moreno *et al.*	2017	HAND	11/6*	48	0.5 (0.3-0.8)^b^	*COWAT, total score* Animals Test, total score TMT B Stroop Interference TOL Total Moves WCST % Error	*0.23 (0.88)^a^* -0.36 (0.77)^a^ -0.30 (1.80)^a^- 0.38 (0.72)^a^ 1.05 (0.81)^a^ 0.35 (0.69)^a^		*↑, NR* ↓, NR ↑, NR ↓, NR ↓, NR ↓, NR
Decloedt *et al.*	2016	HAND	32	24	0.6 - 1.0^x^	*Animals Test, total score* Fruit and Vegetables Test Color Trails Test 2 Stroop WCST	*13.72 (2.96)* *-14.06 (3.05)* 154.82 (45.95) 24.19 (8.25) 41.0 (31.0–87.5)^b^	*14.06 (2.66)* 13.81 (3.18) 143.33 (111.52–169.12)^b^^+^ 26.84 (9.17)^+^ 41.0 (29.5–59.5)^b^	*↑, NR* ↓, NR ↑, NR ↑, NR NC, NR
Kohno *et al.*	2007	Healthy	20	4	0.8	*Word Fluency Test* TMT B	*35.3 (8)* 65.7 (15.9)	*36 (7.8)* 64.3 (22.2)^+^	*↑, NS* ↑, NS

Abbreviations: N = number of participants, mmol/L = millimoles per litre, Li = lithium, BD type 1 = bipolar disorder type 1, mg = milligrams, Comm. = Commission, Om. = Omission, RT = reaction time, ms = milliseconds, NS = not significant, HAND = HIV-associated Neurocognitive Disorders, COWAT = Controlled Oral Word Association Test, TMT B = Trail Making Test, NR = not reported, TOL = Tower of London, WCST = Wisconsin Card-Sorting Test, WAIS = Weschler Adult Intelligence Scale, ↑ = improved, ↓ = declined, NC = no/negligible change.

* = N baseline / N analysis, ^a^ = mean change, ^b^ = median, interquartile range, ^+^ = positive values indicate worsening, - = negative values indicate improvement, ^x^ = where actual mean serum not available, target serum level or dose is reported, ^y^ = mean value in li responders.

Texts in *italics* denote the tests and results on verbal fluency.

In regards of verbal fluency, the mania study found non-significant improvements after 8 weeks of lithium treatment (Small et al., 1986); meanwhile, the two HANDs studies showed mixed results (Decloedt et al., 2016; Munoz-Moreno et al., 2017). Additionally, the healthy volunteer study reported a lack of change in verbal fluency (Kohno et al., 2007) (Table 6).

### Lithium monotherapy and combination therapy

Among the 30 included studies, 25 employed lithium monotherapy while 5 utilised combination therapy with agents, including anticonvulsants (valproate, carbamazepine), antidepressants (nortriptyline), cholinesterase inhibitors (physostigmine) or lecithin (Mardani et al., 2021; Randels et al., 1984; Smith et al., 2010; Stoudemire et al., 1998; Telford and Worrall, 1978). Combination therapy was predominantly employed in affective disorder populations (80% vs 36%), more frequently included non-euthymic patients at baseline (50% vs 11%), had smaller mean sample sizes (*n* = 19.6 vs 24.8) and more variable treatment durations, with 40% employing short protocols (⩽2 weeks) compared to none in the monotherapy group. In the domain of global cognition, combination therapy demonstrated a higher improvement rate (67%; 2/3 studies) compared to monotherapy (25%; 3/12 studies), with the most pronounced benefits observed when lithium was combined with physostigmine (Telford and Worrall, 1978). Combination therapy studies assessed fewer cognitive domains overall; only one combination study evaluated memory (Smith et al., 2010) and one assessed executive function (Mardani et al., 2021), both showing mixed or no significant changes.

### Populations

Across different diagnostic groups, lithium’s cognitive effects exhibited significant heterogeneity. In BDs, improvements were noted in global cognition, processing speed, attention and executive function, particularly when mood stabilisation was achieved (Small et al., 1986). For other/mixed psychotic and affective disorders, findings were less consistent (although clearly this in itself is a heterogeneous population group). For example, a 4-week trial in schizophrenia/schizoaffective disorder reported attention improvements (Small et al., 2003), while mixed affective reversal studies showed declines in memory after lithium reinstatement (Kocsis et al., 1993; Shaw et al., 1987). In neurocognitive disorders, lithium showed promise in improving global cognition and memory, particularly in Alzheimer’s disease and HANDs. Healthy volunteers generally showed improvements in memory and processing speed but declines in attention at high doses (Bell et al., 2005; Calil et al., 1990; Müller-Oerlinghausen et al., 1977). To further elucidate these findings, we summarised the distribution of study outcomes across cognitive domains in Table 7. This table highlights the percentage of studies reporting significant improvements and declines, non-significant changes, and mixed outcomes.

**Table 7.** Summary of outcomes across cognitive domains.

Domain	N studies	N Sig. improvement (%)	N NS improvement (%)	N NS decline (%)	N Sig. decline (%)	N Mixed (%)
Global cognition	15	5 (33.3)	6 (33.3)	1 (6.67)	0	3 (20)
Memory	19	6 (31.5)	7 (36.8)	1 (5.26)	3 (15.79)	2 (10.5)
Processing & psychomotor speed	8	2 (25)	1 (12.5)	3 (37.5)	1 (12.5)	1 (12.5)
Attention	9	2 (22.2)	1 (11.1)	2 (22.2)	2 (22.2)	2 (22.2)
Verbal fluency & executive function	6	0	1 (16.6)	4 (66.7)	0	1 (16.7)

Abbreviations: Sig. = significant, NS = non-significant.

Sig. improvement/decline indicates overall direction with at least one significant test results. NS improvement/decline indicates overall direction without clear or significant reporting. Mixed denotes studies with equal improvement/decline results, regardless of significance or where change was negligible i.e. no direction.

### Risk of bias

Five studies were rated as having a low RoB, 11 moderate and 14 high. The predominant factors influencing these elevated RoB assessments were a lack of intention-to-treat (ITT) analysis undertaken and randomisation procedures (mainly due to studies being non-randomised trials) and insufficiently detailed reporting. Details of the RoB assessment are shown in Supplement 2.

To examine the potential influence of completer-only bias on our findings, we conducted a systematic analysis comparing studies based on their analytical approach: nine studies employed full ITT analysis, four used partial or unclear ITT approaches and 17 relied exclusively on completer-only analyses. Study characteristics were comparable across groups, with ITT studies averaging 26.0 participants over 17.3 weeks and completer-only studies averaging 25.2 participants over 16.5 weeks, with similar population distributions. When comparing cognitive outcomes across analysis types, global cognition improved in half of the ITT studies (2/4) versus one-third of completer-only trials (3/9); in memory, ITT studies showed no improvements, while completer-only and partial-ITT trials reported a significant increase in 3/10 and 2/4 studies, respectively. Processing/psychomotor speed significantly improved in none of the ITT studies (0/2) but in 2/6 completer-only trials; attention followed the same pattern with no ITT gains versus 2/4 completer-only improvements. Executive function and verbal-fluency outcomes were uniformly non-significant across all studies.

## Discussion

This review provides the first comprehensive synthesis of lithium’s effects on cognition across various populations. The findings show considerable heterogeneity within the domains of global cognition, memory, processing speed, psychomotor speed, attention, verbal fluency and executive function: 9 studies reported significant improvements, 6 noted declines, while the remaining 15 studies showed mixed outcomes, with a summary of the overall findings presented in Table 7.

### Cognitive domains

In the domain of global cognition, there was an equal split between studies showing no change and those showing improvements. Three out of four studies reported improvements in individuals with affective disorders; however, it is unclear whether these were due to isolated cognitive impacts, secondary effects related to changes in mood or a combination of both (Small et al., 1986; Stoudemire et al., 1998; Telford and Worrall, 1978). These improvements align with the recent findings of a longitudinal study on BD and a cross-sectional study on amnestic MCI (Burdick et al., 2020; Damiano et al., 2023). Adjusted analyses often showed no significant lithium and concomitant psychotropic drugs correlation, but instances like adding physostigmine injection in one study, which enhanced cholinergic activity in the central nervous system, demonstrated improved global cognition (Telford and Worrall, 1978).

Findings on memory, which were extensively examined in 19 studies, showed a relatively balanced distribution of outcomes (between improvement, worsening and lack of change). Among the four studies reporting improvements, one was of an extremely long duration with participants presenting symptoms at baseline (Small et al., 1986). Another long-term study did not include people with affective disorders, suggesting that the observed effect might be independent of mood stabilisation (Forlenza et al., 2019). The remaining three studies, spanning 2–4 weeks, involved healthy subjects (Bell et al., 2005; Calil et al., 1990; Stip et al., 2000). On the other hand, among the four studies indicating a decline in memory performance, one was similar to the short-term study involving healthy individuals, but the other three yielded slightly different findings resulting from reversal or discontinuation (Christodoulou et al., 1981; Kocsis et al., 1993; Kropf and Müller-Oerlinghausen, 1979; Shaw et al., 1987). The impairment observed is consistent with a previous review and meta-analysis on both clinical and healthy population; however, this was further elucidated in another review which indicated that acute administration of lithium did not appear to impact short-term memory performance in healthy volunteers and that deficits observed with subchronic lithium may be transient (Pachet and Wisniewski, 2003; Tsaltas et al., 2009; Wingo et al., 2009). It is also important to note that memory is a composite construct, and our review did not distinguish between different memory subtypes, which could potentially introduce variability in the results.

Studies on processing and psychomotor speed showed inconsistent results (many of which were non-significant or where significance was not reported), including those using reversal effect (i.e. effects post lithium discontinuation), despite the similar nature of those studies. Moreover, two studies (one healthy volunteers and one mania for 4–8 weeks) had shown a significant improvement in processing speed, and one in mixed affective patients indicated a significant worsening after 2 weeks of lithium reinstatement; however, the limited number of studies in this area further complicates the interpretation of these findings (Calil et al., 1990; Shaw et al., 1987; Small et al., 1986). These mixed findings stand in contrast to the previous findings of narrative review and meta-analysis of a worsening effect of lithium on processing speed (Pachet and Wisniewski, 2003; Wingo et al., 2009). However, it is worth noting that even a mild change in mood could sensitively affect processing speed, making it challenging to disentangle lithium’s effects on cognition from those on mood.

In the domain of attention, findings showed a relatively balanced distribution of outcomes (between lack of change, improvement and worsening). One of the studies showing improvement involved participants who were manic at baseline; thus, the observed improvements may be attributable to the therapeutic intervention of the mood episode (Small et al., 1986). It is also worth noting that the studies that indicated worsening included healthy volunteers within a short duration of intervention (Müller-Oerlinghausen et al., 1977; Stip et al., 2000). These are comparatively consistent with earlier findings, which also did not observe significant effects of lithium on attention observed in the previous meta-analysis and a recent cross-sectional study involving euthymic BD patients (Bersani et al., 2016; Wingo et al., 2009).

The outcomes on verbal fluency and executive function revealed limited evidence supporting substantial conclusions, particularly given the multifaceted nature of executive function. Notably, the study showing improvements in verbal fluency also included subjects who exhibited manic episodes at baseline (Small et al., 1986). Conversely, investigations into executive function predominantly reported a trend of negligible change. This is in line with studies noting preserved executive function in BD patients, particularly those of lithium responders (Rybakowski et al., 2009; Senturk et al., 2007).

### Populations and interventions

In BDs, lithium often improves cognitive functions, particularly when used to stabilise mood (Small et al., 1986). However, these improvements may be confounded by mood stabilisation effects, making it challenging to isolate lithium’s direct cognitive impact. For instance, studies have shown that excellent lithium responders, who experience no affective recurrences during treatment, perform similarly to healthy controls on cognitive tests (Rybakowski et al., 2009; Senturk et al., 2007). Additionally, lithium may reduce the risk of dementia in bipolar patients, potentially through its neuroprotective effects (Hajek et al., 2014; Nunes et al., 2007). For schizophrenia and other affective disorders, the evidence is less robust and more variable. Some studies suggest cognitive benefits, such as improvements in attention and global cognition (Small et al., 2003; Zhuo et al., 2022). However, these findings are not consistent across all studies, and more research is needed to fully understand lithium’s cognitive effects in these populations.

In neurocognitive disorders, lithium shows promise in enhancing global cognition and memory, particularly in Alzheimer’s disease and HANDs. This aligns with lithium’s potential neuroprotective effects, which may involve mechanisms such as GSK-3 inhibition and increased BDNF expression (Chen and Chuang, 1999; Forlenza et al., 2014). Meanwhile, healthy volunteers generally demonstrate improvements in memory and processing speed but declines in attention at high doses (Bell et al., 2005; Calil et al., 1990; Müller-Oerlinghausen et al., 1977). This variability underscores the need for careful consideration of lithium’s cognitive side effects, particularly in populations that may be more susceptible to them, such as older adults or those with pre-existing cognitive impairments (Grandjean and Aubry, 2009).

Our review identified only three studies examining lithium in BD populations despite its primary indication. This scarcity reflects several constraints including restricted placebo controls in maintenance studies given the evidence of treatment-refractory risk after lithium discontinuation, the application of combination therapy in real practice, and historical gaps between establishing mood efficacy and developing standardised cognitive assessments (Berk, 2007; Burdick et al., 2015; Kim et al., 2021; Kupka et al., 2024; Shorter, 2009).

Our analysis of treatment approaches revealed important methodological patterns that inform interpretation of lithium’s cognitive effects. Among the 30 studies, 83.3% employed monotherapy while 16.7% used combination therapy. Combination therapy was preferentially used in affective disorder populations (80% vs 36% in monotherapy) and more frequently included non-euthymic patients (50% vs 11%), suggesting ‘confounding by indication’ where more unwell patients receive more complex treatment regimens (Brookhart et al., 2010; Salas et al., 1999). While combination therapy showed higher improvement rates for global cognition (67% vs 25% in monotherapy), this comparison is limited by small numbers (*n* = 3 studies) and restricted cognitive domain assessment, with only one combination study each evaluating memory or executive function.

These patterns suggest that apparent cognitive differences between monotherapy and combination therapy likely reflect baseline population differences and specific augmenting agents rather than inherent superiority of combination approaches. The clinical reality is that combination therapy has become the rule rather than the exception in BD treatment (Fornaro et al., 2016; Kim et al., 2021). The preferential use of combination therapy in acutely ill patients with greater room for improvement, combined with limited cognitive domain assessment complicates interpretation (Nunes et al., 2011; Vickers and Altman, 2001). This reinforces our review’s central finding: lithium’s cognitive effects remain heterogeneous across populations and methodologies, with treatment complexity adding another layer of variability. Future studies should account for these selection biases when evaluating lithium’s cognitive impact, particularly given the clinical reality that most bipolar patients require combination therapy, making ‘pure’ monotherapy effects increasingly academic in practice.

### Lithium’s side effects and neurotoxicity

From a clinical perspective, lithium’s cognitive effects pose challenges for both patients and clinicians. Patients often report subjective cognitive difficulties, such as memory problems, attention deficits and mental slowing, which can affect daily functioning and medication adherence (Rybakowski, 2018). Interestingly, despite these reported cognitive challenges, our findings revealed an increase overall in memory performance following lithium treatment, which seems to correlate with mood improvements (Small et al., 1986). This discrepancy between subjective experience and objective test performance is important and may be driven by mood-related bias, medication expectations, and patients’ insights or illness parameters in psychiatric populations (Bonnín et al., 2024; Burdick et al., 2005; Martínez-Arán et al., 2005).

Our analysis revealed an important methodological consideration that may partially explain this discrepancy. When comparing studies by analytical approach, a pattern emerged: completer-only studies reported memory improvements while ITT studies showed no memory improvements at all. This suggests that selection bias in completer-only analyses could operate through multiple mechanisms. First, bidirectional attrition excludes both those experiencing cognitive side effects and early non-responders who experience neither mood stabilisation nor cognitive enhancement (Kessing et al., 2007; Rybakowski, 2018). Second, a ‘therapeutic alliance bias’ may influence outcomes, as patients maintaining positive relationships with treatment teams demonstrate better engagement and potentially enhanced motivation during cognitive testing (Martin et al., 2000). Third, the phenomenon of ‘cognitive adaptation’ – whereby initial cognitive complaints resolve with continued treatment – remains captured only in completers, while those experiencing the most severe early effects discontinue before adaptation might occur (Small et al., 2003; Stip et al., 2000). This finding reconciles the conflict between clinical observations of cognitive complaints leading to discontinuation and research reports of cognitive enhancement, suggesting both phenomena coexist in different subpopulations.

Our findings contribute to the ongoing debate regarding lithium’s neuroprotective versus neurotoxic effects. While evidence suggests that higher lithium levels may increase cognitive risks, maintaining lower therapeutic concentrations (0.4–0.6 mmol/L) can, in some cases, reduce these effects without compromising efficacy (Ferensztajn-Rochowiak and Rybakowski, 2023). However, defining an optimal lithium dose remains clinically challenging, as cognitive impairment may also result from other lithium-induced (and dose related) side effects, such as tremor, fatigue and polyuria, which can indirectly affect cognitive function (Dols et al., 2013). Additionally, older adults and individuals with pre-existing cognitive impairments are more vulnerable due to higher serum lithium levels per administered dose (Grandjean and Aubry, 2009). Despite these concerns, our study did not identify a clear dose–outcome relationship, likely due to minimal dosage variation across participants and the reliance on averaged data rather than detailed dose-cognition analyses. Indeed, generally, doses were higher in psychiatric populations than neurocognitive illness populations, but a lack of difference between these may be related to the mood effects (and mood-cognition correlations) in those with affective disorders. This underscores the need for more precise research on lithium’s dose-dependent cognitive effects to optimise its therapeutic use while minimising risks.

### Strengths and limitations

Definitive trends regarding lithium’s cognitive impact remain inconclusive based on the studies reviewed, although several determinants potentially influencing this outcome were identified. The findings within affective disorders are still debated, particularly in delineating lithium’s raw cognitive efficacy from illness-related indicators, such as mood improvement. This complexity is further compounded by the existence of responder subgroups and cognitive reserve clusters within affective illness populations, particularly BD, which adds variability to the observed outcomes (Malhi et al., 2016; Tsapekos et al., 2020). We were limited by the heterogeneity of the included populations, which may have obscured potential lithium-related cognitive effects specific to certain groups. It is also important to note that there was only a limited number of studies in most populations, including those where lithium is most commonly used, such as individuals with BD, where there is a known cognitive aspect to the disorder.

Additionally, most included studies analysed only treatment completers, introducing attrition bias, where individuals with cognitive side effects discontinue treatment. This leads to an overrepresentation of those who tolerate it well – a limitation potentially explaining the gap between clinical observation and research findings (Wingo et al., 2009). Treatment discontinuers potentially represent a cognitive vulnerability subgroup who remain invisible in most research, raising the possibility that completer-only analyses systematically exclude genetically predisposed individuals who might benefit from alternative treatments or modified dosing strategies (Rybakowski, 2018). Future research should prioritise prospective designs including both completers and dropouts to clarify the incidence and the trajectory of cognitive side effects, employing ITT analyses, systematic documentation of discontinuation reasons and statistical methods such as mixed-effects models that account for non-random dropout patterns (Little et al., 2012).

The diverse neuropsychological assessments employed as cognitive batteries increase heterogeneity, particularly considering the sensitivity and specificity of different tests across different cognitive domains. Issues such as practice effects and the sensitivity of measures (e.g. the Mini-Mental State Examination (MMSE) being less sensitive to impairment detection in affective disorders) complicate the interpretation of results (Burdick et al., 2015; Green, 2006; Spering et al., 2012). Additionally, this review included older studies, some of which used cognitive tests that may not fully align with current neuropsychological standards (Telford and Worrall, 1978). However, several included studies incorporated standardised and widely validated cognitive assessments, such as the MCCB, WAIS-III, TMT and ADAS-Cog, which enhance comparability across research findings (Lezak et al., 2012; Strauss et al., 2006). The inclusion of these more recent instruments helps bridge the gap between older and contemporary studies, allowing for a more accurate understanding of cognitive outcomes.

Some studies have also explored the reversal effects of lithium; however, these types of studies are not widely replicated, leaving the exact nature and duration of this effect uncertain. These considerations highlight the challenges in establishing a clear understanding of lithium’s cognitive impact, particularly given the likelihood of study samples being biased due to the tendency of individuals who tolerate lithium well to remain on treatment. To mitigate this limitation, future research should prioritise studies with well-matched groups randomised to receive or not receive lithium, ensuring comparability across populations. Additionally, standardised and sensitive assessment tools, as well as replication studies that consider illness parameters within specific illness populations, are essential to draw definitive conclusions in this area.

This review acknowledges the limitations of the studies examined, particularly the confined sample sizes, the range of cognitive domains and assessments employed, potential biases introduced by non-randomised trials, unclear reporting and participant loss. Additionally, in assessing the RoB, we used overall scores to indicate potential bias, and the ‘Not Applicable’ or ‘Not Reported’ responses were not factored into the overall ratings; this may have impacted the assessment. Another limitation was our inability to assess a dose effect in depth, and it also appeared that shorter studies were less likely to show benefits compared to those of longer duration. There is an overlap where studies involving healthy volunteers were shorter, and cognitive studies in general employed lower doses than those used in affective disorders, which complicates the analysis. The exhaustive inclusion criteria enabled a comprehensive evaluation but also added complexities in the synthesis of the results. Despite these challenges, this review marks the first attempt to provide an updated and extensive investigation of lithium’s effect on human cognition, highlighting research heterogeneity. Our within-subjects analysis controls for individual differences and provides higher statistical power with smaller samples; however, it is susceptible to practice effects and increased participant attrition. While this approach reduces variability related to illness parameters, it limits generalisability compared to a between-subjects design. Incorporating a between-subjects comparison could help identify relevant factors influencing outcome measures (Burdick et al., 2015; Charness et al., 2012; Malhi et al., 2016).

### Further research and clinical implications

Further investigation of lithium’s impact on cognition requires larger randomised controlled trials across distinct populations. Longitudinal studies should disentangle lithium’s cognitive effects from illness-related confounders by examining different illness stages (e.g. pre, onset and during treatment) and subgroup analyses (e.g. different treatment responses, baseline cognitive deficits and subjective complaints alignment) using more updated, standardised cognitive batteries and performance-based functional measures. Additionally, investigating the association between cognitive function and lithium response – specifically comparing excellent responders to non-responders – could provide further insights (Rybakowski and Suwalska, 2010). Given the typically mild and potentially reversible nature of cognitive effects observed in affective disorder studies, a critical focus should be on understanding their functional implications for individuals on long-term lithium. Healthcare providers should address non-adherence driven by fear rather than concrete side effects (Morselli et al., 2003), emphasizing cognitive risks and serum level monitoring. When counselling patients about lithium therapy, clinicians should acknowledge that reported cognitive benefits predominantly reflect the experience of treatment persisters, and discussions should focus on cognitive preservation in the context of mood stabilisation rather than enhancement. For patients with pre-existing cognitive concerns or cognitively demanding occupations, baseline cognitive assessment and early monitoring may identify those at risk for discontinuation and allow for timely dose adjustments or therapeutic alternatives. Ultimately, prioritising resolution of main symptoms should remain the primary therapeutic goal.

Lithium, which has long-standing and wide-ranging evidenced neuropsychiatric benefits, has relatively little research examining specifically its cognitive effects. This systematic review underscores the diverse findings across cognitive domains and populations, highlighting the challenge of interpreting lithium’s cognitive impact. There is a clear call for robust, longitudinal controlled studies with an emphasis on subgroup analysis. Amidst these intricacies, recognising the mild changes without immense cognitive decline lays the groundwork for patients’ education and informed therapeutic decisions involving lithium.

## Supplemental Material

sj-docx-1-jop-10.1177_02698811251371139 – Supplemental material for The effects of lithium on cognition in humans: A systematic review Supplemental material, sj-docx-1-jop-10.1177_02698811251371139 for The effects of lithium on cognition in humans: A systematic review by Talitha Najmillah Sabtiari, Samuel Myrtle, Stelios Orfanos, Allan H. Young and Rebecca Strawbridge in Journal of Psychopharmacology

sj-docx-2-jop-10.1177_02698811251371139 – Supplemental material for The effects of lithium on cognition in humans: A systematic review Supplemental material, sj-docx-2-jop-10.1177_02698811251371139 for The effects of lithium on cognition in humans: A systematic review by Talitha Najmillah Sabtiari, Samuel Myrtle, Stelios Orfanos, Allan H. Young and Rebecca Strawbridge in Journal of Psychopharmacology
