# Supplementary material for: The effects of lithium on cognition in humans: A systematic review
Source: J Psychopharmacol. 2025 Oct 17;39(10):1135–53. doi: 10.1177/02698811251371139 (PMC12572361; doi:10.1177/02698811251371139)
Supplement: sj-docx-1-jop-10.1177_02698811251371139 – Supplemental material for The effects of lithium on cognition in humans: A systematic review [file sj-docx-1-jop-10.1177_02698811251371139.docx]

**Supplement 1 Outline Protocol Changes**

**Protocol Number:** [CRD42023407053](https://www.crd.york.ac.uk/PROSPERO/display_record.php?RecordID=407053)

**Current Approved Protocol:** Version 1 (10 March 2023)

**Amended Protocol:** Version 2 (22 July 2024)

**List of changes between original and amended protocols:**

1. **Searches**

Minor refinements to search terms: removal of “concentration” and “learning”; additions of “executive function”, “verbal fluency” and “processing”.

The final search used was therefore as follows: (LITHIUM and (COGNIT* or NEUROCOGNIT* or MEMORY or EXECUTIVE or LEARNING or ATTENTION or PSYCHOMOTOR or PROCESSING SPEED or LANGUAGE) and (HUMAN or CLINICAL or PARTICIPANT or PATIENT or VOLUNTEER or PEOPLE)).ab, ot, ti.

*Rationale for Change:* To maximise relevant articles from the search while maintaining a search whose results were feasible to review in a timely fashion.

1. **Comparator(s)/control.**

Amended to focus solely on within- subjects comparison between lithium-present and lithium-absent conditions, rather than both within- and between-subjects.

*Rationale for Change:* It became apparent early in the review process that it would not be possible to extract meaningful data from the variety of between-subjects comparisons; focusing on within-subjects comparisons where participants act as their own control can reduce individual variability and illness parameters prone to confound results.

1. **Types of study to be included.**

The protocol was revised from including all quantitative primary research to specifically primary interventional research.

*Rationale for Change:* This was to ensure focus on studies with direct lithium interventions.

1. **Risk of bias (quality) assessment.**

The same tools were used as pre-specified, but were modified to include group comparisons and potential confounders, in addition to the criteria used in the old protocol.

*Rationale for Change:* It became apparent that modified assessments would be required to standardise the evaluation of both randomised and non-randomised study designs.
