# Supplementary material for: The effects of lithium on cognition in humans: A systematic review
Source: J Psychopharmacol. 2025 Oct 17;39(10):1135–53. doi: 10.1177/02698811251371139 (PMC12572361; doi:10.1177/02698811251371139)
Supplement: sj-docx-2-jop-10.1177_02698811251371139 – Supplemental material for The effects of lithium on cognition in humans: A systematic review [file sj-docx-2-jop-10.1177_02698811251371139.docx]

| **No** | **Authors** | **Year** | **Bias domains** | | | | | | | | | **Score** | **NA/NR*** | **Overall Risk** |
| --- | --- | --- | --- | --- | --- | --- | --- | --- | --- | --- | --- | --- | --- | --- |
|  |  |  | **1** | **2** | **3** | **4** | **5** | **6** | **7** | **8** | **9** |  |  |  |
| 1 | **Devanand *et al.*** | 2022 | 1 | 1 | 1 | 1 | 1 | 1 | 1 | 1 | 0.5 | 8.5 | 0 | Low |
| 2 | **Zhuo *et al.*** | 2022 | NA | NA | 0 | 0.5 | 0 | 0 | 0 | 0.5 | 0 | 1 | 2 | High |
| 3 | **Mardani *et al.*** | 2021 | 1 | 0.5 | 0 | 1 | 1 | 1 | 1 | 0 | 1 | 6.5 | 0 | Low |
| 4 | **Forlenza *et al.*** | 2011, 2019 | 1 | 1 | 1 | 1 | 1 | 0.5 | 1 | 1 | 0.5 | 8 | 0 | Low |
| 5 | **Sun *et al.*** | 2019 | NA | NA | 0 | NA | NA | 0.5 | 0 | 1 | 0.5 | 2 | 4 | High |
| 6 | **Munoz-Moreno *et al.*** | 2017 | 0.5 | NR | 1 | 0.5 | 0 | 0 | 1 | 1 | 1 | 5 | 1 | Moderate |
| 7 | **Decloedt *et al.*** | 2016 | 1 | 1 | 1 | 1 | 1 | 1 | 1 | 0.5 | 0.5 | 8 | 0 | Low |
| 8 | **Kellner *et al.*** | 2016 | 1 | 1 | 1 | 0.5 | 0.5 | 1 | 1 | 1 | 0.5 | 7.5 | 0 | Low |
| 9 | **Nunes *et al.*** | 2013 | NR | NR | 1 | 1 | 1 | 0 | 1 | 0.5 | 0.5 | 5 | 2 | Moderate |
| 10 | **Smith *et al.*** | 2010 | 1 | NR | 0 | 0.5 | 1 | 1 | 0.5 | 1 | 0.5 | 5.5 | 1 | Moderate |
| 11 | **Macdonald *et al.*** | 2008 | NA | NA | 1 | NA | 0 | 0 | 0.5 | 1 | 0.5 | 3 | 3 | High |
| 12 | **Hampel *et al.,* Leyhe *et al.*** | 2009 | 1 | 1 | 1 | 0.5 | 1 | 0 | 0 | 1 | 0.5 | 6 | 0 | Moderate |
| 13 | **Schifitto *et al.*** | 2009 | NA | NA | 0 | NA | NA | 0 | 0 | 1 | 1 | 2 | 4 | High |
| 14 | **Kohno *et al.*** | 2007 | NA | NA | 1 | NA | NA | 1 | 0 | 1 | 1 | 4 | 4 | Moderate |
| 15 | **Yucel *et al.*** | 2007 | NA | NA | 1 | NA | NA | 0.5 | 0 | 0 | 1 | 2.5 | 4 | High |
| 16 | **Bell *et al.*** | 2005 | NR | NR | 1 | 1 | 1 | 0.5 | 0.5 | 1 | 1 | 6 | 2 | Moderate |
| 17 | **Small *et al.*** | 2003 | 1 | 0.5 | 1 | 1 | 0.5 | 0 | 1 | 0 | 0 | 5 | 0 | Moderate |
| 18 | **Stip *et al.*** | 2000 | 0.5 | NR | NR | 1 | 1 | 0 | 1 | 1 | 0.5 | 5 | 2 | Moderate |
| 19 | **Stoudemire *et al.*** | 1998 | NR | NR | 1 | 0.5 | 1 | 1 | 1 | 0.5 | 1 | 6 | 2 | Moderate |
| 20 | **Kocsis *et al.*** | 1993 | NA | NA | 0 | 1 | NA | 0 | 0 | 0.5 | 1 | 2.5 | 3 | High |
| 21 | **Calil *et al.*** | 1990 | 0.5 | NR | 1 | 1 | 1 | 0 | 1 | 0.5 | 1 | 6 | 1 | Moderate |
| 22 | **Shaw *et al.*** | 1987, 1986 | NA | NA | 0 | 0.5 | NA | 0 | 0 | 1 | 1 | 2.5 | 3 | High |
| 23 | **Small *et al.*** | 1986 | NR | NR | 0 | 0.5 | 1 | 0 | 0 | 0 | 1 | 2.5 | 2 | High |
| 24 | **Randels *et al.*** | 1984 | NA | NA | 0 | NA | NA | 0 | 0 | 0.5 | 0 | 0.5 | 4 | High |
| 25 | **Smigan *et al.*** | 1983 | NA | NA | 1 | NA | NA | 0 | 0 | 0 | 1 | 2 | 4 | High |
| 26 | **Brinkman *et al.*** | 1982 | NA | NA | NR | 0.5 | NA | 1 | 0 | 0.5 | 0.5 | 2.5 | 4 | High |
| 27 | **Christodoulou *et al.*** | 1981 | NA | NA | 0 | 0.5 | NA | 0 | 1 | 1 | 1 | 3.5 | 3 | High |
| 28 | **Kropf *et al.*** | 1979 | NR | NR | 1 | 1 | 1 | 1 | 0.5 | 0.5 | 1 | 6 | 2 | Moderate |
| 29 | **Telford *et al.*** | 1978 | NA | NA | NR | NA | NA | 0 | 0.5 | 1 | 0.5 | 2 | 5 | High |
| 30 | **Müller-Oerlinghausen *et al.*** | 1977 | NA | NA | NR | NA | 0 | 0 | 0 | 0.5 | 1 | 1.5 | 4 | High |
|  |  |  |  |  |  |  |  |  |  |  |  | *Total scores indicating overall risk:* | | |
|  | 0 = high risk  0.5 = unclear or moderate risk |  |  |  |  |  |  |  |  |  |  | *0-3* |  | *High Risk* |
|  | 1 = low risk |  |  |  |  |  |  |  |  |  |  | *4-6* |  | *Moderate Risk* |
|  |  |  |  |  |  |  |  |  |  |  |  | *7-9* |  | *Low Risk* |

**Supplement 2. Table of Risk of Bias**

Bias domains: 1 = allocation sequence randomized, 2 = allocation sequence concealed, 3 = baseline groups comparable / confounders found and controlled, 4 = groups allocation blinded, 5 = groups equally treated, 6 = intention-to-treat analysis, 7 = appropriate outcome measures, 8 = protocol deviations, 9 = selection of reported results

* number of domains rated as not applicable (NA) or not reported (NR).
